# Low doses of 3-phenyl-lawsone or meglumine antimoniate delivery by tattooing route are successful in reducing parasite load in cutaneous lesions of *Leishmania* (*Viannia*) *braziliensis*-infected hamsters

**DOI:** 10.3389/fcimb.2023.1025359

**Published:** 2023-01-19

**Authors:** Rafaella de Miranda Villarim Meira, Sara Lins da Silva Gomes, Edgar Schaeffer, Thayssa Da Silva, Andréia Carolinne de Souza Brito, Larissa Moreira Siqueira, Job Domingos Inácio, Elmo Eduardo Almeida-Amaral, Alda Maria Da-Cruz, Milla Bezerra-Paiva, Renata Heisler Neves, Luciana Silva Rodrigues, Patricia Maria Lourenço Dutra, Paulo Roberto Ribeiro Costa, Alcides José Monteiro da Silva, Silvia Amaral Gonçalves Da-Silva

**Affiliations:** ^1^ Laboratório de Imunofarmacologia Parasitária, Universidade do Estado do Rio de Janeiro, Rio de Janeiro, Brazil; ^2^ Laboratório de Catálise Orgânica, Universidade Federal do Rio de Janeiro, Rio de Janeiro, Brazil; ^3^ Laboratório de Bioquímica de Tripanosomatídeos, Fundação Oswaldo Cruz, Rio de Janeiro, Brazil; ^4^ Disciplina de Parasitologia, Universidade do Estado do Rio de Janeiro, Rio de Janeiro, Brazil; ^5^ Laboratório Interdisciplinar de Pesquisas Médicas, Fundação Oswaldo Cruz, Rio de Janeiro, Brazil; ^6^ Laboratório de Helmintologia Romero Lascasas Porto, Universidade do Estado do Rio de Janeiro, Rio de Janeiro, Brazil; ^7^ Laboratório de Imunopatologia, Faculdade de Ciências Médicas, Universidade do Estado do Rio de Janeiro, Rio de Janeiro, Brazil; ^8^ Laboratório de Imunologia do Exercício, Universidade do Estado do Rio de Janeiro, Rio de Janeiro, Brazil; ^9^ Laboratório de Química Bioorgânica, Universidade Federal do Rio de Janeiro, Rio de Janeiro, Brazil

**Keywords:** *Leishmania (Viannia) braziliensis*, 3-phenyl-lawsone, chemotherapy, tattooing, subcutaneous, hamster-model

## Abstract

Current therapeutic ways adopted for the treatment of leishmaniasis are toxic and expensive including parasite resistance is a growing problem. Given this scenario, it is urgent to explore treatment alternatives for leishmaniasis. The aim of this study was to evaluate the effect of 3-phenyl-lawsone (3-PL) naphthoquinone on *Leishmania* (*Viannia*) *braziliensis* infection, both *in vitro* and *in vivo*, using two local routes of administration: subcutaneous (higher dose) and tattoo (lower dose). *In vitro* 3-PL showed low toxicity for macrophages (CC_50_ >3200 µM/48h) and activity against intracellular amastigotes (IC_50_ = 193 ± 19 µM/48h) and promastigotes (IC_50_ = 116 ± 26 µM/72h), in which induced increased ROS generation. Additionally, 3-PL up-regulated the production of cytokines such as tumor necrosis factor alpha (TNF-α), monocyte chemotactic protein 1 (MCP-1), interleukin-6 (IL-6) and IL-10 in infected macrophages. However, the anti-amastigote action was independent of nitric oxide production. Treatment of hamsters infected with *L.* (*V.*) *braziliensis* from one week after infection with 3-PL by subcutaneous (25 µg/Kg) or tattooing (2.5 µg/Kg) route, during 3 weeks (3 times/week) or 2 weeks (2 times/week) significantly decreased the parasite load (p<0.001) in the lesion. The reduction of parasite load by 3-PL treatment was comparable to reference drug meglumine antimoniate administered by the same routes (subcutaneous 1mg/Kg and tattoo 0.1mg/Kg). In addition, treatment started from five weeks after infection with 3-PL per tattoo also decreased the parasite load. These results show the anti-leishmanial effect of 3-PL against *L.* (*V.*) *braziliensis* and its efficacy by subcutaneous (higher dose) and tattoo (lower dose) routes. In addition, this study shows that drug delivery by tattooing the lesion allows the use of lower doses than the conventional subcutaneous route, which may support the development of a new therapeutic strategy that can be adopted for leishmaniasis.

## 1 Introduction

American Tegumentary Leishmaniasis (ATL) is the most common clinical form of leishmaniasis ranging from self-healing wounds to severe mucosal tissue damage of the infected individual. Of the total cases of ATL in 2020, 42% were reported by Brazil (Pan American Health Organization, 2021). The available therapies for various forms of leishmaniasis are based on systemic administration of antimonial pentavalent or amphotericin B and pentamidine presenting extensive toxicity, high costs, emerging drug resistance and varied efficacy depending upon the species, symptoms and geographical region (Berman, 2005; Santos et al., 2008; Pimentel et al., 2011; Ponte-Sucre et al., 2017). These limitations encourage the search for new therapeutic alternatives to treat leishmaniasis (Mudavath et al., 2014; Zulfiqar et al., 2017). In Brazil, ATL is caused mostly by species *Leishmania* (*Viannia*) *braziliensis* (Cupolillo et al., 2003; David and Craft, 2009; Ministério da Saúde, 2017) which cause both cutaneous (CL) and mucosal leishmaniasis (ML). Most studies related to this species are performed in humans since mice develop small, self-healing lesions that have limited application. In this sense, the hamster model demonstrates a more appropriate alternative, because when infected with *L.* (*V.*) *braziliensis* it mimics human CL (Gomes-Silva et al., 2013; Ribeiro-Romão et al., 2014; Dutra and da Silva, 2017) and proved to be a suitable model to evaluate antileishmania vaccines (da Silva-Couto et al., 2015) and drugs (Costa et al., 2014). In general, the drug of first choice for treating *L.* (*V.*) *braziliensis* infection is meglumine antimoniate (Glucantime^®^). However, this therapy has several limitations, as high parenteral doses are required, inducing moderate to severe side effects that lead to discontinuation of treatment and contribute to increased parasite resistance (Carvalho et al., 2019). Therefore, the development of new drugs and alternative drug delivery is necessary for the treatment of CL (Zulfiqar et al., 2017; Caridha et al., 2019). Intralesional (IL) pentavalent antimonials have been used for decades for the treatment of CL in the Old World (World Health Organization, 2022). However, in America, this use was recommended in 2013 for patients with single lesions, nursing mothers, and contraindications to systemic treatment (nephropathies, hepatopathies, cardiopathies). It is also contraindicated for lesions larger than 3 cm in diameter, or those located in the head or periarticular areas, and for immunosuppressed patients (Pan American Health Organization, 2013). More recently, Brazil adhered to this recommendation to treat lesions, up to 3 cm in the greatest diameter, at any location except the head and periarticular regions (Ministério da Saúde, 2017).

The use of a modified tattoo device for medical purposes has been utilized for various applications, such as in dermatological treatments (Sadeghinia and Sadeghinia, 2012; Arbache et al., 2018), implantation of glucose monitoring detectors (Yetisen et al., 2019), an indication of tumor location for surgery (Yigit et al., 2022) and DNA vaccines (Oosterhuis et al., 2012; Fotoran et al., 2020). New transdermal drug delivery methods, such as needle-free injectors (NFIs), microneedles, and tattoo devices have been developed and may have advantages over some traditional delivery methods, including the use of low doses and coverage of large areas of skin (Arbache et al., 2019; Mercuri and Rivas, 2021). Shio and collaborators (2014) used a tattoo device to administer a liposomal formulation of oleylphosphocholine to mice infected with *L.* (*L.*) *major* or *L.* (*L.*) *mexicana* and showed the efficacy of this approach.

Naphthoquinones are natural molecules with high biological activity and pharmacological potential due to its redox cycle, which promotes the production of reactive oxygen species (Ferreira et al., 2010; Qiu et al., 2018). There is a variety of natural and synthetic naphthoquinones with antimalarial, antihelminthic, anti-*Trypanosoma* and anti-*Leishmania* activities (Reimão et al., 2012; Hazra et al., 2013; Rezende et al., 2013; Rocha et al., 2013; Lorsuwannarat et al., 2014; Oliveira et al., 2018). Lapachol is a natural naphthoquinone isolated from several plants of the Bignoniaceae family, mainly *Tecoma* and *Tabebuia* species. Several studies demonstrate lapachol as a pharmacological agent (Hussain et al., 2007; Hussain and Green, 2017), with antitumor (Sunassee et al., 2013; Zu et al., 2019), antimicrobial (Oliveira et al., 2010; Souza et al., 2013) and antiparasitic activities (Teixeira et al., 2001; Lima et al., 2004; Salas et al., 2011). Therefore, the study of synthetic lapachol derivates and analogues has been proposed as potential antiparasitic drugs (Lima et al., 2004; Rocha et al., 2013; Barbosa et al., 2014). Lawsone, a 2-hydroxy-1,4-naphthoquinone originally obtained from the henna plant (*Lawsonia inermis*), as well as derivatives molecules, have diverse biological properties such as antitumor, antimicrobial, and antiparasitic action (reviewed by Pradhan et al., 2012; al Nasr et al., 2019).

The aim of this study was to evaluate the activity of the synthetic lapachol derivative 3-phenyl-lawsone (3-PL) against *L.* (*V.*) *braziliensis in vitro*, as well as its therapeutic potential using local routes of administration subcutaneous (higher dose) and tattooing (lower dose) compared with the reference drug meglumine antimoniate, in experimentally infected hamsters.

## 2 Materials and methods

### 2.1 3-phenyl-lawsone

**Figure 1:**
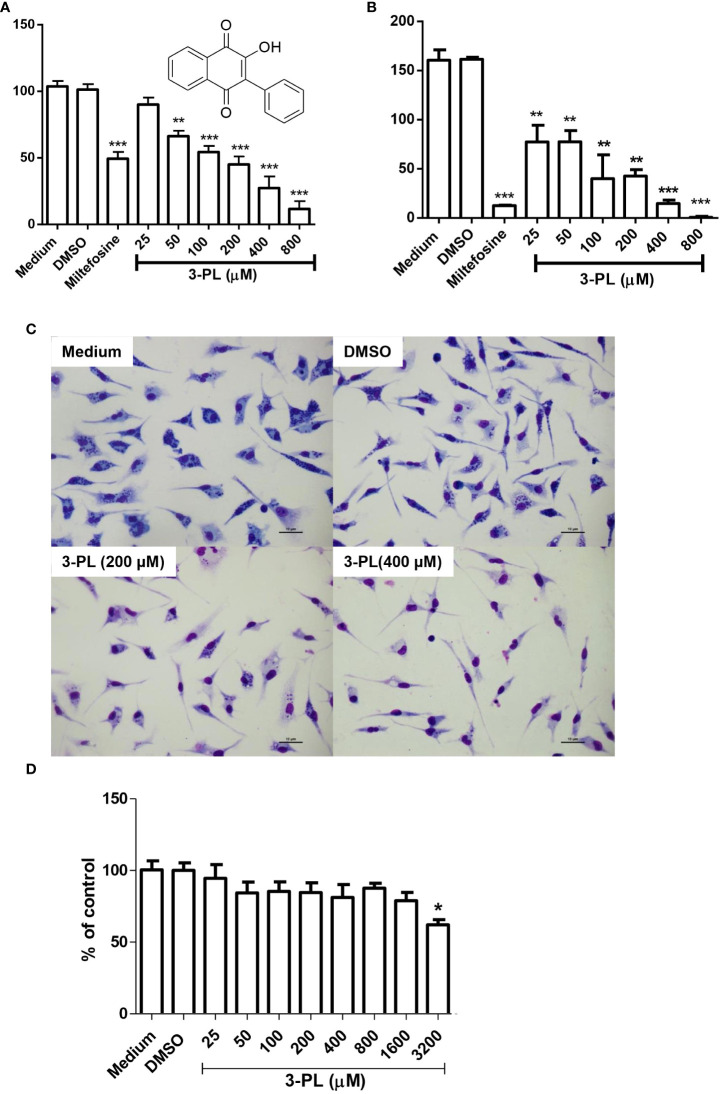
*In vitro* activity of 3-PL against *L. (Viannia) braziliensis* amastigotes and macrophage toxicity. **(A-C)** Murine macrophage monolayers were infected with stationary-phase promastigote of *L.* (*V.*) *braziliensis* in a ratio of 5:1 (parasites/macrophage). Infected macrophages were incubated in an atmosphere of 5% CO2 at 37°C, with RPMI 1640 medium supplemented with 10% FBS for 24h to differentiate amastigotes. Afterwards, the infected monolayers were submitted to treatment with indicated concentrations of 3-PL or 3 µM miltefosine for 48h. **(A)** The infection index was determined by counting at least 200 macrophages and expressed as % of control or **(B)** the treated infected macrophages were washed twice and incubated with Schneider’s medium plus 20% FBS at 28°C for more than 48h and promastigotes were counted. **(C)** Representative light microscopy images of infected macrophage monolayers after 48h of treatment. **(D)** Toxicity on peritoneal murine macrophages treated during 48h at 5% CO_2_/37°C. Results (A,B and D) were presented as means ± SD; n=3. *p < 0.05, **p < 0.01 and ***p < 0.001 in relation to control DMSO.

The lapachol derivative 3-phenyl-lawsone, 3-PL, (**Figure 1A**, insert) was synthesized in the Laboratory of Bioorganic Chemistry of the Federal University of Rio de Janeiro, Brazil by the Suzuki-Miyaura reaction as previously described (Gomes et al., 2017). For assays, the 3-PL was dissolved in DMSO (Sigma Aldrich, St Louis, MO, USA) whose final concentration did not exceed 1%.

### 2.2 Parasites culture

*Leishmania* (*Viannia*) *braziliensis* (MCAN/BR/98/R619) parasites were routinely isolated from hamster lesions and maintained as promastigotes in Schneider’s insect medium (Sigma-Aldrich, St Louis, MO, USA) containing 20% heat-inactivated fetal bovine serum (FBS) (Cultilab, Brazil) and 10 µg/mL gentamicin (Schering-Plough, Kenilworth, New Jersey, USA). The medium was changed weekly and parasites were used until no more than six passages.

### 2.3 Activity on intracellular amastigotes

Peritoneal macrophages from BALB/c mice obtained as described above, were plated (2x10^6^ cells/well) onto glass coverslips placed within the wells of a 24-well culture plate and infected with stationary phase promastigotes of *L.* (*V.*) *braziliensis* at a multiplicity of infection (MOI) of 5:1, in 5% atmosphere of CO_2_ at 37°C, for 4h. The infected monolayers were washed to remove non-internalized parasites and incubated with RPMI 1640 medium supplemented with 10% of FBS in 5% atmosphere of CO_2_ at 37°C, for 24h, to assure the differentiation into amastigotes forms. Then the infected macrophages were treated for 48h with 3-PL (0-800 μM), in 5% atmosphere of CO_2_ at 37°C. The control cells were macrophages incubated with medium or vehicle (DMSO 0.2%) or reference drug miltefosine at 3µM (IC_50_). Controls and 3-PL concentrations were performed in triplicate and the experiment was repeated at least three times. Then, the supernatants were collected and stored at -20°C for further measurement of nitric oxide or cytokine production. The monolayers were then dyed with a fast panoptic stain (Laborclin, Brazil) and amastigotes were quantified in, at least, 200 macrophages per sample. The results were expressed as an infection index (= % infected cells X number of amastigotes/total number of macrophages). The 50% inhibitory concentration (IC_50_) was determined by logarithmic regression analysis using GraphPad Prism 6.

To assess the viability of the remaining amastigotes after 48h of treatment, we investigated the ability of these amastigote to differentiate into promastigotes. Infected macrophage monolayers were washed twice with PBS (heated to 37°C) and incubated with Schneider’s medium and 20% FBS at 28°C for an additional 48h, when promastigotes were counted in a Neubauer chamber.

### 2.4 Cytotoxicity assay to macrophages

The toxicity of 3-PL on mammalian cells was evaluated in peritoneal macrophages from BALB/c mice (6-8 weeks old) by MTT assay (Mosmann, 1983). Resident macrophages were obtained from peritoneal cells of BALB/c mice after intraperitoneal injection of 5 mL of cold RPMI 1640 medium without FBS (Cultilab, Brazil). The peritoneal cells of the mice (4-8 x 10^6^ cells/animal) were pooled and plated in a 96-well culture plate (8x10^5^ cells/well) and after 1h, non-adherent cells were removed. Monolayers of peritoneal macrophages were cultured in triplicate with RPMI 1640 medium supplemented with 10% of FBS and 3-PL over a wide concentration range (0-3200 μM) at 37°C in 5% atmosphere of CO_2_ for 48h. MTT (5 mg/mL) was added (20 μL/well) and the plates were incubated for 3h. The supernatants were removed, formazan crystals were dissolved in DMSO and absorbance was determined in a spectrophotometer at 550 nm using a microplate reader (Epoch - Biotek). The cytotoxic concentration 50% (CC_50_) was determined by logarithmic regression analysis using GraphPad Prism 6.

### 2.5 Nitric oxide production assay

Macrophage production of nitric oxide was measured by nitrite detection using the Griess method (Green et al., 1982). Supernatants from macrophage monolayers were transferred to the plate where the Griess reagent [1% sulfanilamide, added to 0.1% of NEED and 2.5% of phosphoric acid (all purchased from Sigma-Aldrich, St. Louis, MO) was added at a ratio of 1:1 (v/v) and incubated for 10 min, at room temperature. Then, the plate was read in an ELISA reader, at 570 nm. Subsequently, reading values were compared to a standard curve of sodium nitrite (NaNO_2_), and results were expressed in µM of nitrite.

### 2.6 Cytokine production

The evaluation of the production of cytokines in the macrophage supernatant was performed by the Cytometric Bead Array (CBA) method using the Mouse Inflammation kit (BD Bioscience), following the manufacturer’s recommendations. Briefly, undiluted samples were incubated with capture beads labeled with distinct fluorescence intensity conjugated with cytokine-specific antibodies for about 3h in the dark at room temperature, followed by fluorescent detection antibody, and all unbound antibodies were washed away. Data were acquired on a BD fluorescence-activated cell sorting (FACS) FACSCanto II analyzer and results were analyzed using the FCAP Array Software program. Cytokine standard curves ranged from 0-5000 pg/mL.

### 2.7 Activity on promastigotes growth

Promastigotes forms of *L.* (*V.*) *braziliensis* were plated in a 24-well culture plate (2.5 x 10^5^ cells/well) and incubated for 96h at 28°C with 3-PL (0-800 µM) diluted in Schneider’s medium supplemented with 20% of FBS. Controls were promastigotes non-treated or treated with 0.2% DMSO (higher final concentration in this assay) or miltefosine. Controls and 3-PL concentrations were performed in triplicate and the experiment was repeated at least three times. The number of parasites was counted daily with a Neubauer chamber using an optic microscope.

### 2.8 Determination of ROS generation

The evaluation of ROS production was performed on promastigotes of *L.* (*V.*) *braziliensis* treated for 72h with 3-PL (100 and 50 µM, corresponding to IC_50_ and half of the IC_50_), in 5% atmosphere of CO_2_ at 37°C. The controls were incubated with culture medium or 0.025% vehicle DMSO (corresponding to higher final concentration in this assay). After treatment, promastigotes were washed twice in HBSS buffer, adjusted to the concentration of 2x10^6^/mL and incubated in a dark 96-well plate (2 x 10^5^/well) for 20 minutes, with 20 μM of the H2DCFDA probe (2’7’-dichlorodihydrofluorescein diacetate), which in the presence of ROS suffers deacetylation being converted to DCFDA (2’7’-dichlorofluorescein), which accumulated inside the cell was captured by fluorescence. The reading was made in a fluorimeter (Spectra Max M2 - Molecular Devices, Silicon Valley, USA) with wavelengths of 485 nm excitation and emission 530 nm. As a positive control, 2 mM H_2_O_2_ was used. Data was expressed as increased ROS production relative to control (medium).

### 2.9 Therapeutic activity on infected golden hamsters

The antiparasitic effect of 3-PL by local routes (subcutaneous or tattoo) were evaluated using the golden hamsters (*Mesocricetus auratus*) which is a model highly susceptible to infection (Gomes-Silva et al., 2013; Mears et al., 2015). The golden hamsters with 6 to 8 weeks old were infected with 10^6^ promastigotes of *L.* (*V.*) *braziliensis* at stationary phase on the dorsal hind paw, and randomly divided into groups (4-6 animals per group). The treatments started 7 days after the infection and were applied to the infection site by subcutaneous or tattooing routes using two protocols.

Protocol I: Treatments were performed three times a week for three weeks. At the end of the treatment, the animals remained under observation for another three weeks (without treatment), after which they were euthanized. The animals were divided in 4 groups, as follows: group 3-PL sc (n = 6) treated with 3-PL (25 μg/Kg) given subcutaneously; group 3-PL tattoo (n = 6) treated with 3-PL per tattoo (± 2.5 μg/Kg, ranging from a minimum of 1.5 and a maximum of 2.5 μg/Kg); group DMSO sc (n = 6) consisted of animals treated with DMSO vehicle (0.05% in PBS) subcutaneously and group DMSO tattoo (n = 6) treated with DMSO vehicle (0.05% in PBS) per tattoo.

Protocol II: Treatments were performed with two administrations per week for two weeks. At the end of treatment, the animals remained under observation (without treatment) for another week, when they were euthanized. The animals were divided in 6 groups, as follows: group 3-PL sc (n = 4) treated with 3-PL (25 μg/Kg) given subcutaneously; group 3-PL tattoo (n = 4) treated with 3-PL per tattoo (± 2.5 μg/Kg, ranging from a minimum of 1.5 and a maximum of 2.5 μg/Kg); group DMSO sc (n = 4) treated with vehicle DMSO (0.05% in PBS) subcutaneously; group DMSO tattoo (n=4) treated with vehicle DMSO (0.05% in PBS) by tattoo; group Glucantime^®^ sc (n=4) treated subcutaneously with the reference drug meglumine antimoniate (1 mg/Kg of Glucantime^®^, Sanofi-Avenetis Farmaceutica Ltda) and group Glucantime^®^ tattoo (n=4) treated by tattoo (± 0.1 mg/Kg, ranging from a minimum of 0.06 and a maximum of 0.1 mg/Kg, of Glucantime^®^).

In addition, we evaluated the potential of lowest dose 3-PL treatment for tattooing initiated in more chronic stages of infection.

Protocol III: Hamsters were treated from 5 weeks of infection (lesion approximately 2 mm thickness) with 3-PL tattoo (n=3) twice a week for 2 weeks. Control group were DMSO tattoo animals (n=3). At the end of treatment, the animals remained under observation (without treatment) for another 2 weeks, when they were euthanized and parasite load evaluated.

Subcutaneous treatment was performed with 50 μL of the solution using a microsyringe with a 29G needle. Tattoo administration was performed using a professional commercial tattooing machine (White Head, Wujiang Kangtai Medical Instrument, China) and a magnum needle type (5 needs head/0.3 mm thickness each), following the protocol adapted from Shio and collaborators (2014). Each tattooing session consisted of twelve administrations lasting two seconds each, with the five needles oscillating at 60 Hz (60 perforations/second), with a total of 7200 punctures (5x12x2x60). The twelve applications of each session were distributed at the lesion site and we estimate that 3-5 μL of solution were injected per session. Both subcutaneous or tattooing administration were performed under anesthesia using 80 mg/kg Ketamine (Syntec, Brazil) plus 10 mg/kg Xylazine (Syntec, Brazil) by intraperitoneal route.

The lesion thickness were measured weekly with a dial caliper (Mitutoyo, Brazil) and expressed as the difference between the thickness of the infected and uninfected paws. At the end of the experiments, the animals were euthanized and the tissue from the lesions was aseptically removed, ground, and transferred to tubes containing Schneider’s medium plus 20% fetal calf serum. The parasite load was evaluated by limiting dilution assay (Costa et al., 2014). The cell suspension was serially diluted in a 96-well plate, and the parasites were evaluated using limiting dilution analysis after 10 days of culture at 27°C.

### 2.10 Histophatological analysis

Skin fragments were fixed in 10% buffered formalin and processed for paraffin embedding Sections of 3 µm thickness were stained with hematoxylin-eosin (H&E) and observed under light microscopy (Nikon Eclipse 80i, Tokyo, Japan) and images captured in Nikon DS-Ri7 and edited by NIS-Elements AR 3.2 program. The inflammatory parameters were expressed by score, according to Yang and collaborators (2013), from a semi-quantitative analysis according to the intensity of occurrence of each histological parameter. The score was defined as: not observed (score=0); slight observed (score=1); moderately observed (score=2) and full observed (score=3).

### 2.11 Statistical analysis

Each *in vitro* experiment was performed in triplicate in at least two independent experiments and the statistical analysis was based on Student’s t-test. The statistical analysis of the *in vivo* experiments was performed using analysis of variance (ANOVA) and the Tukey *post hoc* test by software GraphPad Prism 6. Differences with a p-value <0.05 were considered as statistically significant. IC_50_ and CC_50_ values were calculated by non-linear regression.

## 3 Results

### 3.1 Anti-amastigote activity of 3-PL and toxicity to mammalian cells

In order to test the antileishmanial the *in vitro* effect of 3-PL against *L.* (*V.*) *braziliensis*, we utilized the intracellular amastigote form of the parasite. Treatment of infected macrophages with 3-PL for 48h showed a significant dose-dependent reduction of the infection index (**Figures 1A, B**). The IC_50_ values were estimated at 193 ± 19 μM. To evaluate the survival of the amastigotes remaining after 3-PL treatment, we investigated the ability of these amastigotes to differentiate into promastigotes. The monolayers of infected and treated macrophages were washed and reincubated with Schneider’s medium plus 20% fetal bovine serum at 28°C for another 48h, and the promastigotes were then counted (**Figure 1B**). The results showed that after treatments with 3-PL or miltefosine the remaining amastigotes lost the capacity to differentiate into promastigotes in relation to untreated control. Although the treatment at 25 µM did not decrease the number of intracellular amastigotes (**Figure 1A**), their capacity to differentiate into promastigotes was compromised (**Figure 1B**). Furthermore, although after treatment with 800 µM 3-PL there was still a certain number of intracellular amastigotes in macrophages (**Figure 1A**) they totally lost the ability to differentiate into promastigotes (**Figure 1B**).

**Figure 1C** displays the appearance of the culture of infected macrophages under the conditions: untreated (medium), treated with vehicle (DMSO), and 3-PL in the concentration range IC_50_ (200 μM) and 2-fold IC_50_ (400 μM) for intracellular amastigote. In **Figure 1D**, we see that 3-PL has low toxicity to mice peritoneal macrophages, with a significant reduction in cell viability only from 3200 μM (38% inhibition). Therefore, the CC_50_ value is above 3200 μM and the estimated selectivity index (CC_50_/IC_50_) is greater than 16.

### 3.2 Nitric oxide and cytokine production by infected macrophage

**Figure 2:**
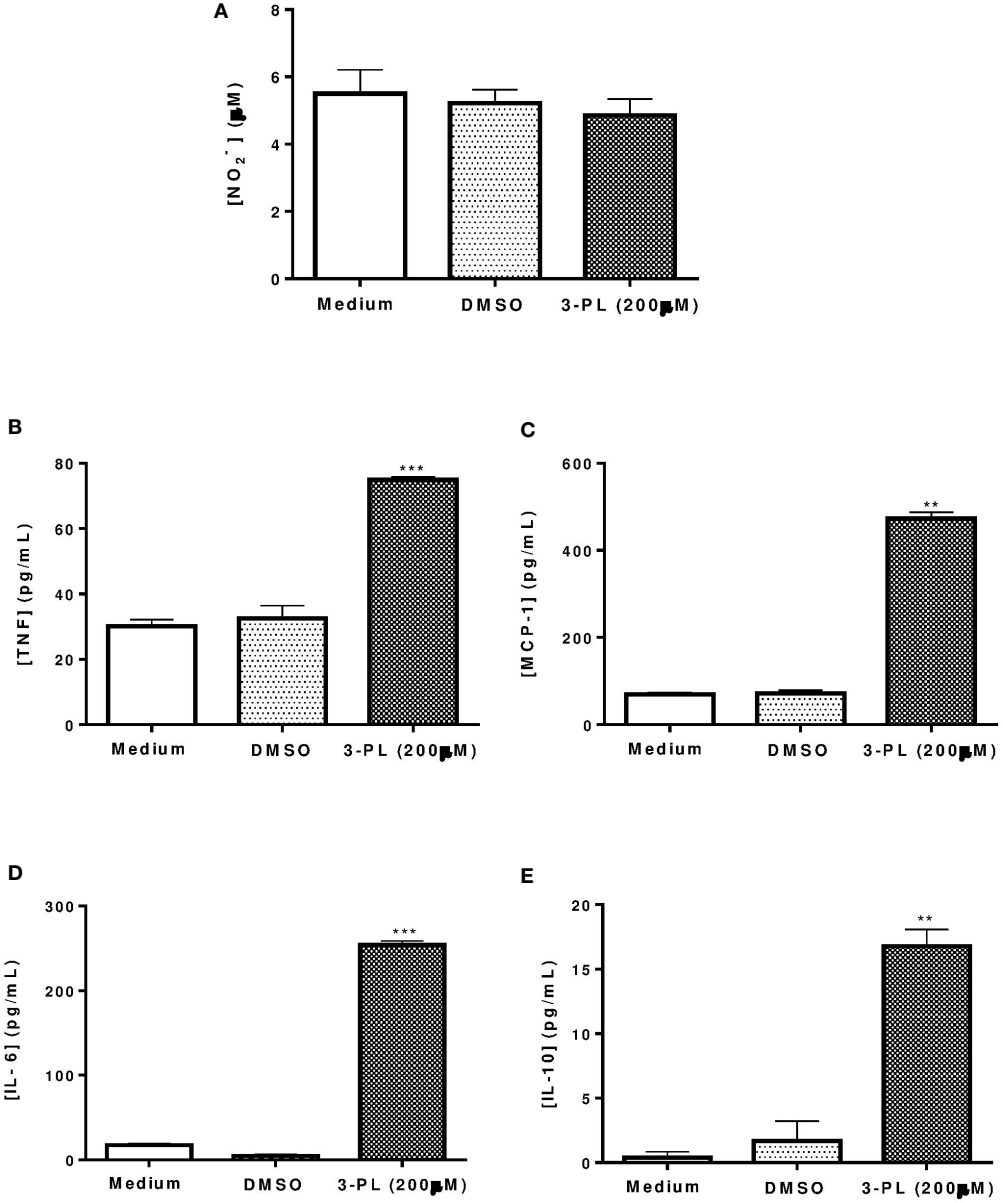
Production of nitric oxide and cytokines by infected macrophages treated with 3-PL. The mediators were measured in the supernatant of macrophages infected with *L.* (*V.*) *braziliensis* after 48h of treatment with 200 µM 3-PL. **(A)** Nitric oxide concentration was evaluated by the Griess method. **(B-E)** Cytokines TNF-α, MCP-1, IL-6 and IL-10 were measured by the Cytometric Bead Array (CBA) method. The data of nitrite (µM) and cytokines (pg/mL) concentration were expressed as the means ± SD, (n=3). **p < 0.01 and ***p < 0.001 in relation to control DMSO.

To assess whether the anti-amastigote activity of 3-PL was associated with host cell activation, we measured nitric oxide and cytokine levels in supernatants from infected macrophages treated with IC_50_ range concentration (200 μM) for 48h. The results showed that nitric oxide was not altered by 3-PL treatment (**Figure 2A**), however, the production of cytokines TNF-α, MCP-1, IL-6, and IL-10 were significantly increased (**Figures 2B–E**).

### 3.3 Anti-promastigote toxicity

**Figure 3:**
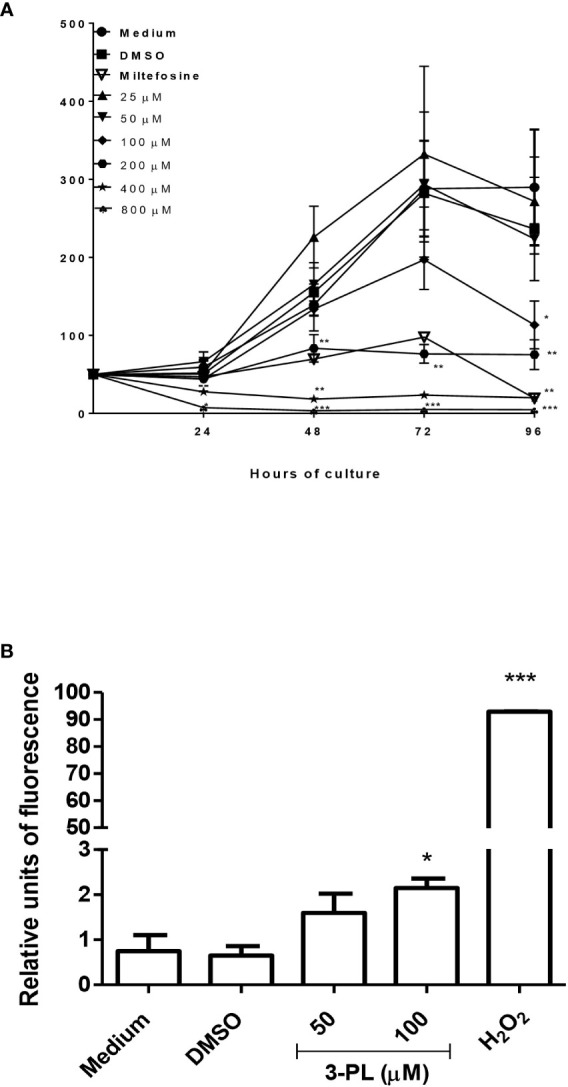
Evaluation of the direct toxic effect of 3-PL on the parasite. Promastigotes were cultured in the presence of indicated concentrations of 3-PL or 6µM miltefosine for 96h at 28°C. **(A)** Promastigote growth was assessed by daily counting in a Neubauer chamber. **(B)** Promastigote ROS generation detected by H2DCFDA probe after treatment with 100 µM 3-PL for 72h. Hydrogen peroxide (H_2_0_2_) was used as a positive control for ROS detection. Results were presented as means ± SD, n=3. *p < 0.05, **p < 0.01 and ***p < 0.001 in relation to control DMSO.

To evaluate the direct effect of 3-PL on parasites, we used the promastigote forms. The 3-PL reduced proliferation of *L.* (*V.*) *braziliensis* promastigote forms in a time- and dose-dependent manner, inhibiting significantly the growth of the parasite from a concentration of 100 µM. In the first 24h, the higher concentrations of 3-PL (400 and 800 µM) promoted the death of the parasites. The IC_50_ was estimated at 116 ± 26 µM for 72h (**Figure 3A**). In order to evaluate the cytotoxic effect of 3-PL on the parasite, we treated the promastigotes for 72h with a concentration in the IC_50_ range (100 µM) or with half the IC_50_ (50 µM) and analyzed the production of ROS by the H2DCFDA probe. Promastigotes treated with 3-PL significantly increased ROS generation (**Figure 3B**) compared to the controls (Medium or DMSO).

### 3.4 *In vivo* therapeutic effects of treatment with high (subcutaneous) and low (tattoing) doses 3-PL in *Leishmania* (*V.*) *braziliensis-*infected hamsters

**Figure 4:**
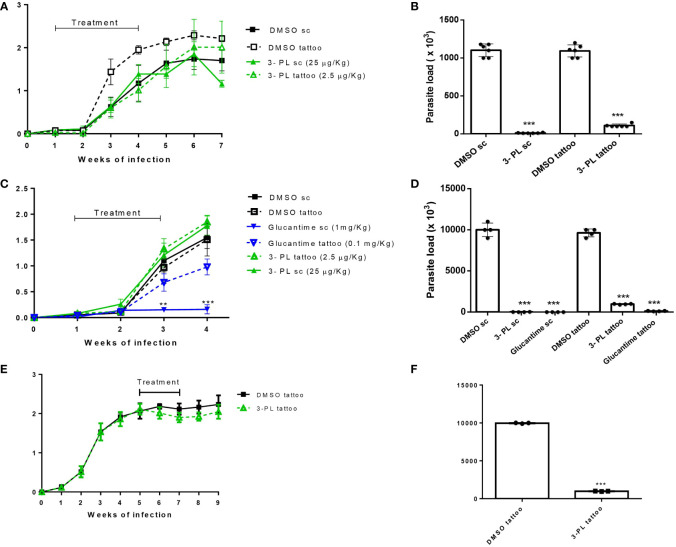
Therapeutic activity of 3-PL administered subcutaneously or tattooing in hamsters infected with *L.* (*V.*) *braziliensis.* Hamsters golden (3-6/group) were infected with 10^6^ promastigotes of *L.* (*V.*) *braziliensis* in the dorsal hind paw and treated from 7 days **(A-D)** or from 5 weeks after infection **(E,F)** with vehicle DMSO 0.5% in PBS (DMSO sc or DMSO tattoo); 3-PL by subcutaneous (3-PL sc, 25 µg/Kg) or tattooing (3-PL tattoo, 2.5 µg/Kg); Glucantime^®^ subcutaneous (Glucantime^®^ sc, 1mg/Kg or tattooing (Glucantime^®^ tattoo, 0.1 mg/Kg) routes. **(A)** Treatment during 3 weeks (3 times/week= 9 doses). **(C, E)** Treatment during 2 weeks (2 times/week= 4 doses). Lesion thickness **(A, C, E)**, expressed as the difference between the thickness of the infected and uninfected paws, were presented as means ± SD, and parasite load in skin paw was determined in the final experiment by limiting dilution analysis **(B, D, F)**; n=4-6; **p < 0.01 and ***p < 0.001 in relation to respective DMSO control (sc or tattoo route).

The therapeutic effect of 3-PL in hamsters infected with *L.* (*V.*) *braziliensis* was evaluated subcutaneously (high doses) or tattooing (low doses) using two treatment protocols. A first approach, protocol I, we treated the animals for three weeks (3 times a week) and followed the lesion for more three weeks without treatment, when we then quantified the parasitic load (**Figures 4A, B**). Treatment with 3-PL subcutaneously or tattooing did not reduce the thickness of the lesion (**Figure 4A**). However, both treatment routes significantly reduced the parasite load (**Figure 4B**) compared to the control groups.

In an attempt to minimize the contribution of probable inflammatory process induced by the local administration on the lesion thickness, a second protocol was performed with reduced time and number of administrations (protocol II). In this protocol, the parasite load determined one week after the end of treatment and included groups treated with the reference drug, meglumine antimoniate (Glucantime^®^). As in the protocol I, we did not observe a decreased thickness of the lesion in the groups treated with 3-PL (subcutaneous or tattoing routes) (**Figure 4C**). However, the parasite load significantly decreased in both 3-PL, subcutaneous or tattooing routes (**Figure 4D**). The treatment with Glucantime^®^ by subcutaneous route, significantly reduces lesion thickness (**Figure 4C**) and parasite load (**Figure 4D**). On the other hand, the treatment of animals with Glucantime^®^ by tattooing did not reduce the thickness of the lesion, however significantly decreased the parasitic load (**Figures 4C, D**). These data show that the administration of both 3-PL and Glucantime^®^ by tattoo, at a dose approximately 10 times lower compared to the subcutaneous route, was able to significantly reduce the parasite load in the lesion, however without reducing the lesion size. Likewise, when we started treating infected animals from the fifth week of infection (lesions about 2 mm thickness) with 3-PL via tattooing for two weeks (**Figure 4E**), there was a significant reduction in the parasite load (**Figure 4F**), but without reducing the thickness of the lesions.

### 3. 5 Histopathological analysis of the lesions

**Figure 5:**
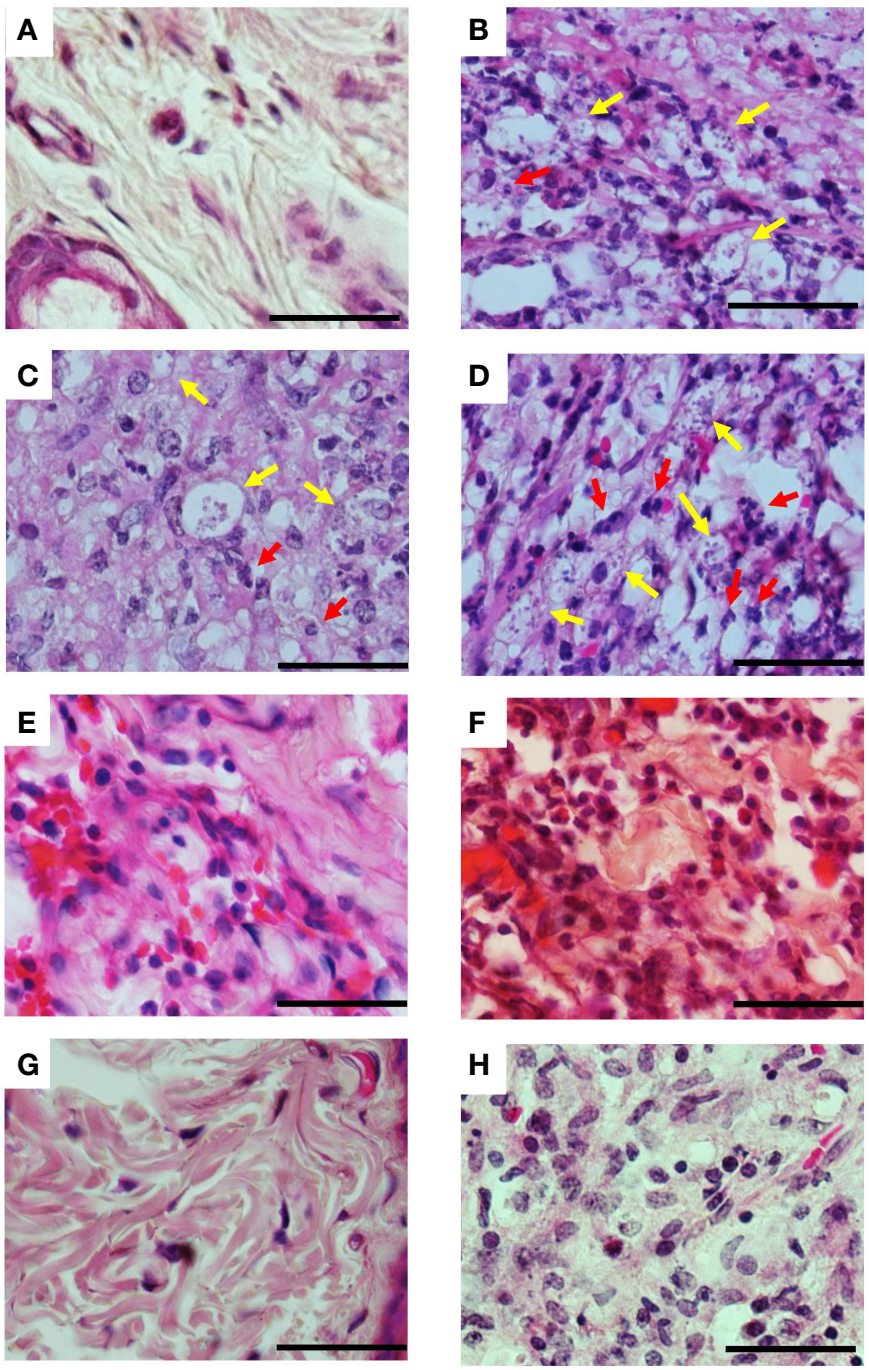
Histopathological aspects of lesions of infected hamsters treated with 3-PL. Golden hamsters (n = 4) infected in the dorsal hind paw with *L.* (*V.*) *braziliensis* were treated with 3-PL or reference drug Glucantime^®^ from one week of infection for two weeks and histological analysis of the lesions was performed by H&E staining. **(A)** Uninfected; **(B)** Infected untreated; **(C)** DMSO sc; **(D)** DMSO tattoo; **(E)** 3-PL sc; **(F)** 3-PL tattoo; **(G)** Glucantime^®^ sc; **(H)** Glucantime^®^ tattoo. Data show representative pictures of each group. Yellow arrow: intracellular amastigotes; Red arrow: neutrophils. 1000x magnification, scale bar = 25 µm.

**Figure 6:**
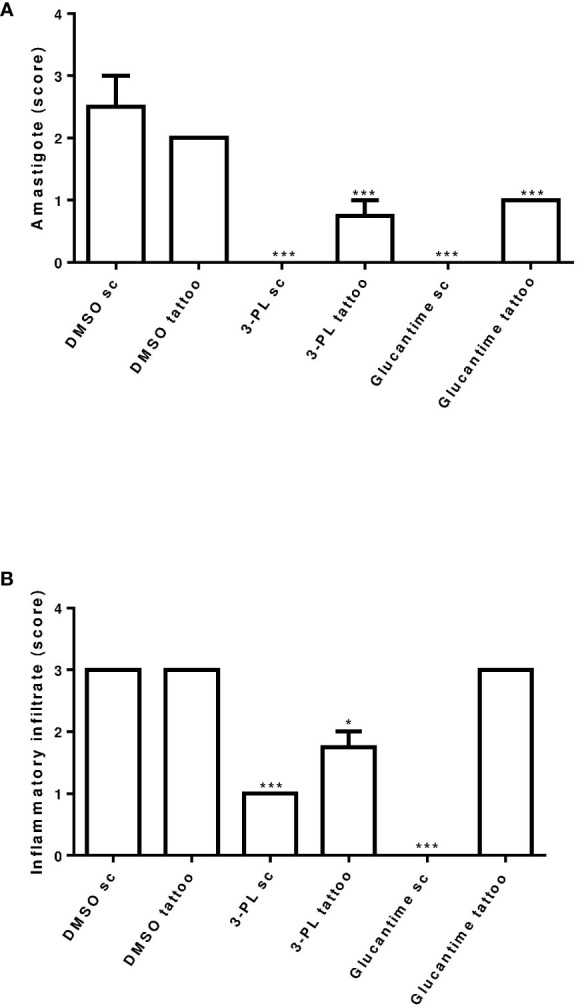
Semi-quantitative analysis of histopathological aspects of hamster skin lesions. Skin lesion sections of hamsters infected with *L.* (*V.*) *braziliensis* treated or not (as indicated) from 7 days of infection for two weeks were stained with H&E and analyzed under light microscopy. In **(A)** presence of amastigote and in **(B)** presence of inflammatory infiltrate. Results were expressed by scoring. The score value was determined as not observed (score = 0), little observed (score = 1), moderately observed (score = 2) and highly observed (score = 3). **p < 0.01 and ***p < 0.001 in relation to respective DMSO control group (sc or tattoo route). *p ≤ 0.05.

Histopathological analysis of the lesions was performed in the groups treated with protocol II (**Figure 5**). In **Figure 5A**, we show the normal appearance of the paw skin of an uninfected hamster. In **Figure 5B**, the infected hamster lesion presents the typical histopathological pattern with an inflammatory infiltrate composed of macrophages showing intracytoplasmic vacuoles containing amastigotes and rare neutrophils. In the lesion of animals treated with 3-PL, both, subcutaneous or tattooing (**Figures 5E, F**, respectively), we observed a predominantly mononuclear inflammatory infiltrate composed of macrophages, lymphocytes, and plasma cells, in addition to rare neutrophils and parasites. In lesions of animals that received 3-PL subcutaneously (**Figure 5E**), the inflammatory infiltrate is more discreet and more localized than in the tattooing route (**Figure 5F**), while amastigotes were not observed. The lesions of the animals treated with Glucantime^®^ subcutaneously (**Figure 5G**) present epidermis, dermis, and a portion of muscle tissue similar in appearance to the histology of the paw tissue of an uninfected animal (**Figure 5A**). Animals treated with Glucantime^®^ *via* tattooing (**Figure 5H**) present lesion tissue with a mixed inflammatory infiltrate (neutrophils, eosinophils, macrophages, and lymphocytes) and rare amastigotes. In **Figure 6**, we show a semi-quantitative analysis using a scoring system for the presence of amastigotes and inflammatory infiltrate in the skin lesion. In hamsters treated with 3-PL or reference drug (Glucantime^®^) by tattooing, amastigotes were rarely observed, while in subcutaneous treatment amastigotes were absent (**Figure 6A**). The inflammatory infiltrate was discreetly observed in animals treated subcutaneously with 3-PL, while in animals treated with Glucantime^®^ by the same route it was not observed (**Figure 6B**). We observed frequent inflammatory infiltrate in the lesions of all groups of animals treated by the tattooing route. However, in the group treated with 3-PL this finding was significantly lower than in the controls groups, including the group treated with Glucantime^®^ (**Figure 6B**).

## 4 Discussion

This study showed an *in vitro* and *in vivo* antileishmania effect of synthetic 3-PL against *L. (V.) braziliensis*. This naphthoquinone presented dose-dependent activity *in vitro*, as on promastigotes (IC_50_ = 116 ± 26 µM, 72h) as well intracellular amastigotes (IC_50_ = 193 ± 19 μM, 48h) forms of *L.* (*V.*) *braziliensis.* These data corroborate with the previous report from our group, that has shown the *in vitro* effect of 3-PL against *Leishmania* (*L.*) *amazonensis*, in both promastigotes (IC_50_ = 85 μM, 72h) and intracellular amastigotes (IC_50_ = 25 μM, 72h) forms (Gomes et al., 2017). The ability of 3-PL to have an effect against species of the different subgenus of the *Leishmania* (subgenus *Leishmania* and *Viannia*), makes it even more promising. The differences between the IC_50_ values obtained in this study for *L. (V.) braziliensis* and those reported for *L. (L.) amazonensis* is expected due to the susceptibility variations between different subgenera and species of *Leishmania*, as well as the experimental conditions used. We established the IC_50_ of 3-PL to *L.* (*V.*) *braziliensis* amastigotes at 48h, while IC_50_ to *L.* (*L.*) *amazonensis* was made at 72h (Gomes et al., 2017). The antileishmanial activity of lapachol, the naphthoquinone precursor molecule of 3-PL, presented wide values of IC_50_ dependent on *Leishmania* strain and experimental conditions. For example, the lapachol IC_50_ for intracellular amastigote of *L.* (*L.*) *amazonensis* was variable from 191 µM/48h (Araújo et al., 2019) to 250 µg/mL at 72h (corresponding to 1 mM/72h) (Costa et al., 2017).

The antileishmanial activity of naphthoquinones has been recognized (Croft et al., 1992; Murray and Hariprashad, 1996; Teixeira et al., 2001; Garnier et al., 2007; Reimão et al., 2012), including synthetic molecules derived from lapachol also show action on *L.* (*V.*) *braziliensis* (Costa et al., 2014) and *L.* (*L.*) *amazonensis* (Cunha-Junior et al., 2011; Ribeiro et al., 2013). The generation of ROS and the modulation of redox signaling are properties of naphthoquinones related to the structural modification in the scaffold (Qiu et al., 2018). As to the mode of action, we observed that the antileishmanial effect of 3-PL was related to increased ROS production and DNA fragmentation (data not shown) of *L.* (*V.*) *braziliensis* promastigotes. Several naphthoquinones are capable of inducing apoptotic death in tumor cells (Wei et al., 2017; Liu et al., 2018; de Almeida et al., 2021) and protozoa (Corrêa et al., 2009; Anjos et al., 2016). Induction of ROS production and DNA fragmentation by quinones and naphthoquinones has also been described in *Leishmania* sp. (Ribeiro et al., 2013; Awasthi et al., 2016). Previous studies from our group showed that pterocarpanquinone LQB-118, a lapachol-pterocarpan derivative, induces ROS production, DNA fragmentation, and cellular death by apoptosis-like on both *L.* (*L.*) *amazonensis* (Ribeiro et al., 2013) and *L.* (*V.*) *braziliensis* (Costa et al., 2014). Furthermore, to induce oxidative stress by ROS, naphthoquinones can act to inhibit topoisomerase. This combination of effects contributes to its important antitumor action, which is part of the mechanism of some naphthoquinone derivatives approved and used clinically against cancer (Qiu et al., 2018). Molecular modeling studies have suggested that 1,4-naphthoquinones tethered to 1,2,3-1H-triazoles have a potential antitumor action mechanism related to inhibition of topoisomerase and/or hPKM2 activity leading to induced microtubule disorganization (Chipoline et al., 2020). Bis-lawsone analogues showed antileishmanal effect associeted with DNA topoisomerase-I inhibition of the parasite (Sharma et al., 2014). The new lawsone derivatives presented the anticancer activity associated with ROS formation and antiparasitic action on *Trypanosoma brucei brucei* related to deformation of the microtubule cytoskeleton (Mahal et al., 2017). Although the increase in ROS may be related to the induction of observed DNA fragmentation (data not shown), we cannot rule out the inhibition of the parasite’s topoisomerase as part of the mechanism of action of 3-PL. Further investigations about the anti-leishmanial mechanism of 3-PL should be carried out. We observed that 3-PL inhibits the multiplication of intracellular amastigotes and also the ability of the remaining amastigotes to differentiate into promastigotes, suggesting an irreversible toxic effect. It is reasonable to assume that the 3-PL toxic mechanism demonstrated for the promastigote could extend to intracellular amastigote forms.

In addition to the direct effect on the parasite, antileishmanial drugs can activate the macrophage to kill intracellular amastigotes (Muniz-Junqueira and de Paula-Coelho, 2008; Ghosh et al., 2013). Our results show that 3-PL modulates infected macrophages by increasing their production of the cytokines TNF-α, IL-6, MCP-1, and IL-10. However, anti-amastigote activity was not related to nitric oxide production. Similar to our results with 3-PL, da Costa-Silva and collaborators (2017) observed that nanoliposomal buparvaquone, a hydroxynaphthoquinone, increases the production of cytokines TNF-α, MCP-1, IL-6 and IL-10 by *Leishmania*-infected macrophages without altering nitric oxide production. Some naphthoquinones have action to prevent the production of nitric oxide (Cheng et al., 2008; Pinho et al., 2011) by inhibition of iNOS protein expression through the downregulation of MAPKqNF-kappaB signaling (Cheng et al., 2008). Furthermore, the increased levels we observed of the anti-inflammatory cytokine IL-10, which downregulates inducible nitric oxide synthase (iNOS) in macrophages, may have contributed to preventing NO production.

The antileishmanial therapeutic potential of 3-PL was investigated for the first time in this study. The evaluation was performed by local routes in hamsters infected with *L.* (*V.*) *braziliensis*. When infected with *L.* (*V*)*. braziliensis*, hamsters develop lesions very similar to those observed in humans, as well as the course of the infection, being considered a good model for therapeutic studies (Gomes-Silva et al., 2013; Mears et al., 2015; Dutra and da Silva, 2017). In the present study, treatment with 3-PL was performed in the lesion site by subcutaneous or tattooing routes. Using two treatment protocols where animals were treated from one week of infection for three or two weeks (Protocols I and II), treatment with 3-PL by both routes, subcutaneously or by tattooing, was able to significantly reduce the parasite load in the lesion. In the same way, when the treatment was initiated in the most chronic phase of the infection (5 weeks of infection, 2 mm thick lesion), protocol III, the administration of 3-PL tattoo for 2 weeks was able to significantly reduce the parasite load. Histopathological data also corroborate this finding and showed a reduction in the number of amastigotes in the tissue. It is important to emphasize that the dose of 3-PL administered in tattooing (± 2.5 µg/Kg) was approximately ten times lower compared to the subcutaneous (25 µg/Kg) route, and even so the molecule was able to reduce the parasite load. Subcutaneous treatment with the reference meglumine antimoniate, Glucantime^®^, has been applied in CL (Carvalho et al., 2019; Rodrigues et al., 2020) and our data confirm the effectiveness of this route of administration using 1mg/Kg. Interestingly, in addition to the well-documented subcutaneous route, lower dose Glucantime^®^ administered by tattooing (± 0.1 mg/Kg) was also effective in reducing the parasite load. This first example of tattoo-mediated pentavalent antimonial delivery may open new therapeutic interventions in the treatment of CL.

Treatment using the delivery drugs directly in skin lesions has been shown to be important in dermatological therapy (Arbache et al., 2018). Novel transdermal methods for drug delivery and vaccination provide a higher immunological response, drug dose sparing, and reduction in pain, which improves patient compliance (Mercuri and Rivas, 2021). Skin tattooing is a new approach to drug delivery (Arbache et al., 2018; Mercuri and Rivas, 2021), intradermal immunization, including DNA vaccination (van de Wall et al., 2015; Fotoran et al., 2020). In the only published study using tattooing as a drug administration route for experimental CL, the anti-*Leishmania* molecule, oleylphosphocholine, was injected in a liposomal formulation to treat mice infected by *L.* (*L.*) *major* or *L.* (*L.*) *mexicana* (Shio et al., 2014). Liposomal formulation increasing treatment efficacy since these particles are prone to ingestion by phagocytic cells such as macrophages (Bruni et al., 2017). In the present study, 3-PL was solubilized in DMSO and administered diluted in PBS and proved to be active. New formulations of 3-PL can be considered for further studies in order to reduce the number of administrations and local inflammation.

The local route of administration may have contributed to the maintenance of inflammation and thickness of the lesions, since it has been shown that there is an inflammatory response caused by tissue damage produced by the injection needle (Gomes-Silva et al., 2013; Mac-Daniel et al., 2014). Our histopathological data corroborate this data, showing the presence of inflammatory infiltrate in the tissue of the lesion in animals treated subcutaneously or tattooing, which probably contributes to not decreasing the thickness of the lesions. We used a total of 4 doses of treatment (twice a week/2 weeks), which must have provided more tissue damage and persistent inflammation. However, the inflammatory infiltrate was more intense in the lesions of the groups of animals treated with tattoos compared to those treated subcutaneously. A histological study evaluating inflammation from ink tattooing on mice skin showed that acute inflammation started at 12h, decreasing its incidence on day 14 (Gopee et al., 2005). In our study, the histopathological analysis was performed one week after the last tattoo session and showed the presence of an inflammatory process. On the other hand, the local inflammation promoted by tattooing may contribute to the induction of an important immune response mediated by T cells, as observed in studies with DNA tattoo vaccines (Platteel et al., 2017; Fotoran et al., 2020; Bakker et al., 2021). In studies of the hamster model infected with *L. (V.) braziliensis* carried out by Ribeiro-Romão and collaborators (2014) and Paiva and collaborators (2021), the lower parasite load generates a more benign course of infection, without systemic involvement and associated with the expression of a more balanced cytokine network. Therefore, terapeutic intervention in the early stages of infection, when the immune response is being mounted by the host, may favor the best outcome of the disease. It is possible to assume that the reduction in the parasite load promoted by the treatment with 3-PL in the initial stages of infection (from seven days after infection) has allowed a more balanced production of cytokines and triggered a more benign course of the disease. Therefore, in addition to the antiparasitic effect of 3-PL, the possible induction of a cellular immune response by the tattoo could have contributed to the great reduction of the parasite load in the animals treated by this route of administration. Furthermore, even when the lesion was already well established, treatment with 3-PL tattooing was also able to reduce the parasite load, demonstrating the promising antileishmanial potential of this naphthoquinone.

In future studies, we may include a longer post-treatment follow-up with 3-PL, allowing the verification of inflammation regression, lesion evolution, and cytokine expression. In addition, we intend to further explore the effect of 3-PL treatment initiated in the more chronic phases of the infection, as well as to investigate the efficacy of combination therapy with reference drugs.

## 5 Conclusions

In this study, we demonstrate the antileishmanial activity of 3-PL against *L. (V.) braziliensis* associated with a direct toxic effect on the parasite involving induction of ROS production and modulation of macrophage cytokines. Using the hamster model of infection, 3-PL has shown efficacy in significantly reducing the parasite load when administered in low doses subcutaneously and by tattooing. Additionally, this study also showed, for the first time, the activity of the reference drug Glucantime^®^ administered by tattooing. Drug administration by tattooing uses small volumes and can be useful in reducing the dose and toxicity of drugs. The dataset gathered in this study indicates that 3-PL has pronounced effects on *L.* (*V.*) *braziliensis* and deserves further preclinical investigations. Therefore, the data presented in this study may contribute to expanding treatment approaches for CL.

## Data availability statement

The original contributions presented in the study are included in the article/supplementary material. Further inquiries can be directed to the corresponding author.

## Ethics statement

The animal study was reviewed and approved by Ethics Committee on Animal Use (CEUA) of the Instituto de Biologia Roberto Alcantara Gomes of the Universidade do Estado do Rio de Janeiro-UERJ, by the number protocol CEUA/051/2017.

## Author contributions

RM conducted experiments and contributed to the writing of the manuscript. SG, ES, TS, AB, LS, JI and MP conducted the experiments. EA-A, AC, RN, LR, PD, PC and AS contributed to the execution and discussion the experiments. SS conducted orientation, experimentation and conducted to the writing of the manuscript. All authors contributed to the article and approved the submitted version.

## References

[B1] al Nasr I. Jentzsch J. Winter I. Schobert R. Ersfeld K. Koko W. S.. (2019). Antiparasitic activities of new lawsone mannich bases. Arch. Pharm. (Weinheim) 352, 1900128. doi: 10.1002/ardp.201900128 31536649

[B2] Anjos D.O.d. Alves E. S. S. Gonçalves V. T. Fontes S. S. Nogueira M. L. Suarez-Fontes A. M.. (2016). Effects of a novel β–lapachone derivative on *Trypanosoma cruzi* : Parasite death involving apoptosis, autophagy and necrosis. Int. J. Parasitol. Drugs Drug Resist. 6, 207–219. doi: 10.1016/j.ijpddr.2016.10.003 27770751 PMC5078628

[B3] Araújo I. A. C. de Paula R. C. Alves C. L. Faria K. F. de Oliveira M. M. Mendes G. G.. (2019). Efficacy of lapachol on treatment of cutaneous and visceral leishmaniasis. Exp. Parasitol. 199, 67–73. doi: 10.1016/j.exppara.2019.02.013 30797783

[B4] Arbache S. Mattos E. da C. Diniz M. F. Paiva P. Y. A. Roth D.. (2019). How much medication is delivered in a novel drug delivery technique that uses a tattoo machine? Int. J. Dermatol. 58, 750–755. doi: 10.1111/ijd.14408 30828798

[B5] Arbache S. Roth D. Steiner D. Breunig J. Michalany N. S. Arbache S. T.. (2018). Activation of melanocytes in idiopathic guttate hypomelanosis after 5-fluorouracil infusion using a tattoo machine: Preliminary analysis of a randomized, split-body, single blinded, placebo controlled clinical trial. J. Am. Acad. Dermatol. 78, 212–215. doi: 10.1016/j.jaad.2017.08.019 29241792

[B6] Awasthi B. P. Kathuria M. Pant G. Kumari N. Mitra K. (2016). Plumbagin, a plant-derived naphthoquinone metabolite induces mitochondria mediated apoptosis-like cell death in *Leishmania donovani*: an ultrastructural and physiological study. Apoptosis 21, 941–953. doi: 10.1007/s10495-016-1259-9 27315817

[B7] Bakker N. A. M. Rotman J. van Beurden M. Zijlmans H. J. M. van Ruiten M. Samuels S.. (2021). HPV-16 E6/E7 DNA tattoo vaccination using genetically optimized vaccines elicit clinical and immunological responses in patients with usual vulvar intraepithelial neoplasia (uVIN): a phase I/II clinical trial. J. Immunother. Cancer 9, 1–13. doi: 10.1136/jitc-2021-002547 PMC8330588 34341131

[B8] Barbosa M. I. F. Corrêa R. S. de Oliveira K. M. Rodrigues C. Ellena J. Nascimento O. R.. (2014). Antiparasitic activities of novel ruthenium/lapachol complexes. J. Inorg Biochem. 136, 33–39. doi: 10.1016/j.jinorgbio.2014.03.009 24727183

[B9] Berman J. (2005). Recent developments in leishmaniasis: Epidemiology, diagnosis, and treatment. Curr. Infect. Dis. Rep. 7, 33–38. doi: 10.1007/s11908-005-0021-1 15610669

[B10] Bruni N. Stella B. Giraudo L. Pepa C.d. Gastaldi D. Dosio F. (2017). Nanostructured delivery systems with improved leishmanicidal activity: a critical review. Int. J. Nanomed. 12, 5289–5311. doi: 10.2147/IJN.S140363 PMC5536235 28794624

[B11] Caridha D. Vesely B. van Bocxlaer K. Arana B. Mowbray C. E. Rafati S.. (2019). Route map for the discovery and pre-clinical development of new drugs and treatments for cutaneous leishmaniasis. Int. J. Parasitol. Drugs Drug Resist. 11, 106–117. doi: 10.1016/j.ijpddr.2019.06.003 31320296 PMC6904839

[B12] Carvalho S. H. Frézard F. Pereira N. P. Moura A. S. Ramos L. M. Q. C. Carvalho G. B.. (2019). American Tegumentary leishmaniasis in Brazil: a critical review of the current therapeutic approach with systemic meglumine antimoniate and short-term possibilities for an alternative treatment. Trop. Med. Int. Health 24, 380–391. doi: 10.1111/tmi.13210 30681239

[B13] Cheng Y. W. Chang C. Y. Lin K. L. Hu C. M. Lin C. H. Kang J. J. (2008). Shikonin derivatives inhibited LPS-induced NOS in RAW 264.7 cells *via* downregulation of MAPK/NF-κB signaling. J. Ethnopharmacol. 120, 264–271. doi: 10.1016/j.jep.2008.09.002 18835347

[B14] Chipoline I. C. da Fonseca A. C. C. da Costa G. R. M. de Souza M. P. Rabelo V. W.-H. de Queiroz. (2020). Molecular mechanism of action of new 1,4-naphthoquinones tethered to 1,2,3-1H-triazoles with cytotoxic and selective effect against oral squamous cell carcinoma. Bioorg. Chem. 101, 103984. doi: 10.1016/j.bioorg.2020.103984 32554278

[B15] Corrêa G. Vilela R. Menna-Barreto R. F. S. Midlej V. Benchimol M. (2009). Cell death induction in *Giardia lamblia*: Effect of beta-lapachone and starvation. Parasitol. Int. 58, 424–437. doi: 10.1016/j.parint.2009.08.006 19703583

[B16] Costa E. V. S. Brígido H. P. C. Silva J. V. Coelho-Ferreira M. R. Brandão G. C. Dolabela M. F. (2017). Antileishmanial activity of *Handroanthus serratifolius* (Vahl) s. grose (Bignoniaceae). Evidence-Based Complementary Altern. Med. 2017, 1–6. doi: 10.1155/2017/8074275 PMC5329664 28286535

[B17] Costa L. Pinheiro R. O. Dutra P. M. L. Santos R. F. Cunha-Júnior E. F. Torres-Santos E. C.. (2014). Pterocarpanquinone LQB-118 induces apoptosis in *Leishmania* (*Viannia*) *braziliensis* and controls lesions in infected hamsters. PloS One 9, e109672. doi: 10.1371/journal.pone.0109672 25340550 PMC4207686

[B18] Croft S. L. Hogg J. Gutteridge W. E. Hudson A. T. Randall A. W. (1992). The activity of hydroxynaphthoquinones against *Leishmania donovani* . J. Antimicrobial Chemother. 30, 827–832. doi: 10.1093/jac/30.6.827 1289357

[B19] Cunha-Junior E. F. Pacienza-Lima W. Ribeiro G. A. Netto C. D. Canto-Cavalheiro M. M. da Silva. (2011). Effectiveness of the local or oral delivery of the novel naphthopterocarpanquinone LQB-118 against cutaneous leishmaniasis. J. Antimicrobial Chemother. 66, 1555–1559. doi: 10.1093/jac/dkr158 21531758

[B20] Cupolillo E. Brahim L. R. Toaldo C. B. Paes de Oliveira-Neto M. de Brito M. E. F. Falqueto A.. (2003). Genetic polymorphism and molecular epidemiology of *Leishmania* (*Viannia*) *braziliensis* from different hosts and geographic areas in Brazil. J. Clin. Microbiol. 41, 3126–3132. doi: 10.1128/JCM.41.7.3126-3132.2003 12843052 PMC165365

[B21] da Costa-Silva T. A. Galisteo A. J. Jr. Lindoso J. A. L. Barbosa L. R. S. Tempone A. G. (2017). Nanoliposomal buparvaquone immunomodulates *Leishmania infantum*-infected macrophages and is highly effective in a murine model. Antimicrob. Agents Chemother. 61, 1–15. doi: 10.1128/AAC.02297-16 PMC5365673 28167544

[B22] da Silva-Couto L. Ribeiro-Romão R. P. Saavedra A. F. da Silva Costa Souza B. L. Moreira O. C. Gomes-Silva A.. (2015). Intranasal vaccination with leishmanial antigens protects golden hamsters (*Mesocricetus auratus*) against *Leishmania* (*Viannia*) *braziliensis* infection. PloS Negl. Trop. Dis. 9, 1–7. doi: 10.1371/journal.pntd.0003439 PMC4287559 25569338

[B23] David C. v. Craft N. (2009). Cutaneous and mucocutaneous leishmaniasis. Dermatol. Ther. 22, 491–502. doi: 10.1111/j.1529-8019.2009.01272.x 19889134

[B24] de Almeida P. D. O. Jobim G.d. S.B. Ferreira C. C. d. S. Bernardes L. R. Dias R. B. Sales C. B. S.. (2021). A new synthetic antitumor naphthoquinone induces ROS-mediated apoptosis with activation of the JNK and p38 signaling pathways. Chem. Biol. Interact. 343, 1–13. doi: 10.1016/j.cbi.2021.109444 33939975

[B25] Dutra P. M. L. da Silva S. A. G. (2017). “Experimental models for trypanosomatids infection,” in Different aspects on chemotherapy of trypanosomatids. Eds. Leon L. Torres-Santos E. C. (New York: Nova Science Publishers, Inc), 39–57.

[B26] Ferreira S. B. Gonzaga D. T. G. Santos W. C. Araújo K. G. de L. Ferreira V. F. (2010). ß-lapachone: Medicinal chemistry significance and structural modifications. Rev. Virtual Química 2, 140–160. doi: 10.5935/1984-6835.20100013

[B27] Fotoran W. L. Kleiber N. Glitz C. Wunderlich G. (2020). A DNA vaccine encoding *Plasmodium falciparum* PfRH5 in cationic liposomes for dermal tattooing immunization. Vaccines (Basel) 8, 619. doi: 10.3390/vaccines8040619 33092277 PMC7711581

[B28] Garnier T. Mantyla A. Jarvinen T. Lawrence J. Brown M. Croft S. (2007). *In vivo* studies on the antileishmanial activity of buparvaquone and its prodrugs. J. Antimicrobial Chemother. 60, 802–810. doi: 10.1093/jac/dkm303 17715126

[B29] Ghosh M. Roy K. Roy S. (2013). Immunomodulatory effects of antileishmanial drugs. J. Antimicrobial Chemother. 68, 2834–2838. doi: 10.1093/jac/dkt262 23833177

[B30] Gomes S. L. S. Militão G. C. G. Costa A. M. Pessoa C. Ó. Costa-Lotufo L. Cunha-Junior E. F.. (2017). Suzuki-Miyaura coupling between 3-iodolawsone and arylboronic acids. synthesis of lapachol analogues with antineoplastic and antileishmanial activities. J. Braz. Chem. Soc. 28, 1573–1584. doi: 10.21577/0103-5053.20160326

[B31] Gomes-Silva A. Valverde J. G. Ribeiro-Romão R. P. Plácido-Pereira R. M. Da-Cruz A. M. (2013). Golden hamster (*Mesocricetus auratus*) as an experimental model for *Leishmania* (*Viannia*) braziliensis infection. Parasitology 140, 771–779. doi: 10.1017/S0031182012002156 23369503

[B32] Gopee N. Cui Y. Olson G. Warbritton A. R. Miller B. J. Couch L. H.. (2005). Response of mouse skin to tattooing: use of SKH-1 mice as a surrogate model for human tattooing. Toxicol. Appl. Pharmacol. 209, 145–158. doi: 10.1016/j.taap.2005.04.003 15913690

[B33] Green L. C. Wagner D. A. Glogowski J. Skipper P. L. Wishnok J. S. Tannenbaum S. R. (1982). Analysis of nitrate, nitrite, and [_15_N]nitrate in biological fluids. Anal. Biochem. 126, 131–138. doi: 10.1016/0003-2697(82)90118-X 7181105

[B34] Hazra S. Ghosh S. das Sarma M. Sharma S. Das M. Saudagar P.. (2013). Evaluation of a diospyrin derivative as antileishmanial agent and potential modulator of ornithine decarboxylase of *Leishmania donovani* . Exp. Parasitol. 135, 407–413. doi: 10.1016/j.exppara.2013.07.021 23973194

[B35] Hussain H. Green I. R. (2017). Lapachol and lapachone analogs: a journey of two decades of patent research(1997-2016). Expert Opin. Ther. Pat. 27, 1111–1121. doi: 10.1080/13543776.2017.1339792 28586252

[B36] Hussain H. Krohn K. Ahmad V. U. Miana G. A. Green I. R. (2007). Lapachol: An overview. Arkivoc 2007, 145–171. doi: 10.3998/ark.5550190.0008.204

[B37] Lima N. M. Correia C. S. Leon L. L. Machado G. M. Madeira M. de F.. (2004). Antileishmanial activity of lapachol analogues. Mem Inst Oswaldo Cruz 99, 757–761. doi: 10.1590/S0074-02762004000700017 15654435

[B38] Liu C. Shen G.-N. Luo Y.-H. Piao X.-J. Jiang X.-Y. Meng L.-Q.. (2018). Novel 1,4-naphthoquinone derivatives induce apoptosis *via* ROS-mediated p38/MAPK, akt and STAT3 signaling in human hepatoma Hep3B cells. Int. J. Biochem. Cell Biol. 96, 9–19. doi: 10.1016/j.biocel.2018.01.004 29326072

[B39] Lorsuwannarat N. Piedrafita D. Chantree P. Sansri V. Songkoomkrong S. Bantuchai S.. (2014). The *in vitro* anthelmintic effects of plumbagin on newly excysted and 4-weeks-old juvenile parasites of *Fasciola gigantica* . Exp. Parasitol. 136, 5–13. doi: 10.1016/j.exppara.2013.10.004 24157317

[B40] Mac-Daniel L. Buckwalter M. R. Berthet M. Virk Y. Yui K. Albert M. L.. (2014). Local immune response to injection of *Plasmodium* sporozoites into the skin. J. Immunol. 193, 1246–1257. doi: 10.4049/jimmunol.1302669 24981449

[B41] Mahal K. Ahmad A. Schmitt F. Lockhauserbäumer J. Starz K. Pradhan R.. (2017). Improved anticancer and antiparasitic activity of new lawsone mannich bases. Eur. J. Med. Chem. 126, 421–431. doi: 10.1016/j.ejmech.2016.11.043 27912173

[B42] Mears E. R. Modabber F. Don R. Johnson G. E. (2015). A review: The current *In vivo* models for the discovery and utility of new anti-leishmanial drugs targeting cutaneous leishmaniasis. PloS Negl. Trop. Dis. 9, e0003889. doi: 10.1371/journal.pntd.0003889 26334763 PMC4559374

[B43] Mercuri M. Rivas D. F. (2021). Challenges and opportunities for small volumes delivery into the skin. Biomicrofluidics 15, 011301. doi: 10.1063/5.0030163 33532017 PMC7826167

[B44] Ministério da Saúde (2017). Manual de vigilância da leishmaniose tegumentar, manual de vigilância da leishmaniose tegumentar. 2nd ed (Brasília: Ministério da Saúde).

[B45] Mosmann T. (1983). Rapid colorimetric assay for cellular growth and survival: Application to proliferation and cytotoxicity assays. J. Immunol. Methods 65, 55–63. doi: 10.1016/0022-1759(83)90303-4 6606682

[B46] Mudavath S. L. Talat M. Rai M. Srivastava O. N. Sundar S. (2014). Characterization and evaluation of amine-modified graphene amphotericin b for the treatment of visceral leishmaniasis: *In vivo* and *in vitro* studies. Drug Des. Devel Ther. 8, 1235–1247. doi: 10.2147/DDDT.S63994 PMC4159315 25214767

[B47] Muniz-Junqueira M. I. de Paula-Coelho V. N. (2008). Meglumine antimonate directly increases phagocytosis, superoxide anion and TNF-α production, but only *via* TNF-α it indirectly increases nitric oxide production by phagocytes of healthy individuals, *in vitro* . Int. Immunopharmacol. 8, 1633–1638. doi: 10.1016/j.intimp.2008.07.011 18692597

[B48] Murray H. W. Hariprashad J. (1996). Activity of oral atovaquone alone and in combination with antimony in experimental visceral leishmaniasis. Antimicrob. Agents Chemother. 40, 586–587. doi: 10.1128/AAC.40.3.586 8851575 PMC163162

[B49] Oliveira R. A. S. Azevedo-Ximenes E. Luzzati R. Garcia R. C. (2010). The hydroxy-naphthoquinone lapachol arrests mycobacterial growth and immunomodulates host macrophages. Int. Immunopharmacol. 10, 1463–1473. doi: 10.1016/j.intimp.2010.08.023 20837170

[B50] Oliveira L. F. G. Souza-Silva F. de Côrtes L. M. Cysne-Finkelstein L. Pereira M.C. de S. de Oliveira J. F.O.. (2018). Antileishmanial activity of 2-Methoxy-4H-spiro-[naphthalene-1,2′-oxiran]-4-one (Epoxymethoxy-lawsone): A promising new drug candidate for leishmaniasis treatment. Molecules 23, 864. doi: 10.3390/molecules23040864 29642584 PMC6017818

[B51] Oosterhuis K. van den Berg J. H. Schumacher T. N. Haanen J. B. A. G. (2012). DNA Vaccines and Intradermal Vaccination by DNA Tattooing. Curr. Top Microbiol. Immunol. 351, 221–250. doi: 10.1007/82_2010_117 21107792

[B52] Paiva M. B. Ribeiro-Romão R. P. Resende-Vieira L. Braga-Gomes T. Oliveira M. P. Saavedra A. F.. (2021). A cytokine network balance influences the fate of *Leishmania* (*Viannia*) *braziliensis* infection in a cutaneous leishmaniasis hamster model. Front. Immunol. 12. doi: 10.3389/fimmu.2021.656919 PMC8281932 34276650

[B53] Pan American Health Organization (2013). Leishmaniasis en las Américas. Recomendaciones para el tratamiento. 1st ed., ed. Pan American Health Organization (Washington, D.C.: Pan American Health Organization).

[B54] Pan American Health Organization (2021) Leishmanioses: informe epidemiológico das américas. pan American health organization. Available at: https://iris.paho.org/handle/10665.2/51742 (Accessed July 28, 2022).

[B55] Pimentel M. I. F. Baptista C. Rubin É.F. Vasconcellos É.de C.F. Lyra M. R. de Matos Salgueiro M.. (2011). Leishmaniose cutânea Americana causada pela *Leishmania* (*Viannia*) *braziliensis* resistente ao antimoniato de meglumina e com boa resposta terapêutica à pentamidina: Relato de um caso. Rev. Soc. Bras. Med. Trop. 44, 254–256. doi: 10.1590/S0037-86822011000200026 21552747

[B56] Pinho B. R. Sousa C. Valentão P. Andrade P. B. (2011). Is nitric oxide decrease observed with naphthoquinones in LPS stimulated RAW 264.7 macrophages a beneficial property? PloS One 6, 1–9. doi: 10.1371/journal.pone.0024098 PMC3162593 21887376

[B57] Platteel A. C. M. Henri S. Zaiss D. M. Sijts A. J. A. M. (2017). Dissecting antigen processing and presentation routes in dermal vaccination strategies. Vaccine 35, 7057–7063. doi: 10.1016/j.vaccine.2017.10.044 29079107

[B58] Ponte-Sucre A. Gamarro F. Dujardin J. C. Barrett M. P. López-Vélez R. García-Hernández R.. (2017). Drug resistance and treatment failure in leishmaniasis: A 21st century challenge. PloS Negl. Trop. Dis. 11, 1–24. doi: 10.1371/journal.pntd.0006052 PMC5730103 29240765

[B59] Pradhan R. Dandawate P. Vyas A. Padhye S. Biersack B. Schobert R.. (2012). From body art to anticancer activities: perspectives on medicinal properties of henna. Curr. Drug Targets 13, 1777–1798. doi: 10.2174/138945012804545588 23140289

[B60] Qiu H.-Y. Wang P.-F. Lin H.-Y. Tang C.-Y. Zhu H.-L. Yang Y.-H. (2018). Naphthoquinones: A continuing source for discovery of therapeutic antineoplastic agents. Chem. Biol. Drug Des. 91, 681–690. doi: 10.1111/cbdd.13141 29130595

[B61] Reimão J. Q. Colombo F. A. Pereira-Chioccola V. L. Tempone A. G. (2012). Effectiveness of liposomal buparvaquone in an experimental hamster model of *Leishmania* (*L.*) *infantum chagasi* . Exp. Parasitol. 130, 195–199. doi: 10.1016/j.exppara.2012.01.010 22281156

[B62] Rezende L. C. D. Fumagalli F. Bortolin M. S. de Oliveira M. G. de Paula M. H. de Andrade-Neto V. F.. (2013). *In vivo* antimalarial activity of novel 2-hydroxy-3-anilino-1,4-naphthoquinones obtained by epoxide ring-opening reaction. Bioorg. Med. Chem. Lett. 23, 4583–4586. doi: 10.1016/j.bmcl.2013.06.033 23850202

[B63] Ribeiro G. A. Cunha-Junior E. F. Pinheiro R. O. da-Silva S. A. G. Canto-Cavalheiro M. M. da Silva A. J. M.. (2013). LQB-118, an orally active pterocarpanquinone, induces selective oxidative stress and apoptosis in *Leishmania amazonensis* . J. Antimicrobial Chemother. 68, 789–799. doi: 10.1093/jac/dks498 23288404

[B64] Ribeiro-Romão R. P. Moreira O. C. Osorio E. Y. Cysne-Finkelstein L. Gomes-Silva A. Valverde J. G.. (2014). Comparative evaluation of lesion development, tissue damage, and cytokine expression in golden hamsters (*Mesocricetus auratus*) infected by inocula with different *Leishmania* (*Viannia*) *braziliensis* concentrations. Infect. Immun. 82, 5203–5213. doi: 10.1128/IAI.02083-14 25287925 PMC4249292

[B65] Rocha M. N. Nogueira P. M. Demicheli C. de Oliveira L. G. da Silva M. M. Frézard F.. (2013). Cytotoxicity and *In vitro* antileishmanial activity of antimony (V), bismuth (V), and tin (IV) complexes of lapachol. Bioinorg Chem. Appl. 2013, 1–7. doi: 10.1155/2013/961783 PMC3677620 23781165

[B66] Rodrigues B. C. Ferreira M. F. Barroso D. H. da Motta J. O. C. de Paula C. D. R. Porto C.. (2020). A retrospective cohort study of the effectiveness and adverse events of intralesional pentavalent antimonials in the treatment of cutaneous leishmaniasis. Int. J. Parasitol. Drugs Drug Resist. 14, 257–263. doi: 10.1016/j.ijpddr.2020.11.002 33285343 PMC7723996

[B67] Sadeghinia A. Sadeghinia S. (2012). Comparison of the efficacy of intralesional triamcinolone acetonide and 5-fluorouracil tattooing for the treatment of keloids. Dermatologic Surg. 38, 104–109. doi: 10.1111/j.1524-4725.2011.02137.x 22093096

[B68] Salas C. O. Faundez M. Morello A. Diego Maya J. XXXA. Tapia R. (2011). Natural and synthetic naphthoquinones active against *Trypanosoma cruzi*: An initial step towards new drugs for chagas disease. Curr. Med. Chem. 18, 144–161. doi: 10.2174/092986711793979779 21110810

[B69] Santos D. O. Coutinho C. E. R. Madeira M. F. Bottino C. G. Vieira R. T. Nascimento S. B.. (2008). Leishmaniasis treatment - a challenge that remains: A review. Parasitol. Res. 103, 1–10. doi: 10.1007/s00436-008-0943-2 18389282

[B70] Sharma G. Chowdhury S. Sinha S. Majumder H. K. Kumar S. V. (2014). Antileishmanial activity evaluation of bis-lawsone analogs and DNA topoisomerase-I inhibition studies. J. Enzyme Inhib Med. Chem. 29, 185–189. doi: 10.3109/14756366.2013.765413 23534930

[B71] Shio M. T. Paquet M. Martel C. Bosschaerts T. Stienstra S. Olivier M.. (2014). Drug delivery by tattooing to treat cutaneous leishmaniasis. Sci. Rep. 4, 1–7. doi: 10.1038/srep04156 PMC3932479 24561704

[B72] Souza M. A. Johann S. dos Santos Lima L. A. R. Campos F. F. Mendes I. C. Beraldo H.. (2013). The antimicrobial activity of lapachol and its thiosemicarbazone and semicarbazone derivatives. Mem Inst Oswaldo Cruz 108, 342–351. doi: 10.1590/S0074-02762013000300013 23778660 PMC4005582

[B73] Sunassee S. N. Veale C. G. L. Shunmoogam-Gounden N. Osoniyi O. Hendricks D. T. Caira M. R.. (2013). Cytotoxicity of lapachol, β-lapachone and related synthetic 1,4-naphthoquinones against oesophageal cancer cells. Eur. J. Med. Chem. 62, 98–110. doi: 10.1016/j.ejmech.2012.12.048 23353747

[B74] Teixeira M. J. de Almeida Y. M. Viana J. R. Holanda Filha J. G. Rodrigues T. P. Prata J. R. C. Jr.. (2001). *In vitro* and *in vivo* leishmanicidal activity of 2-Hydroxy-3-(3-methyl-2-butenyl)-1,4-naphthoquinone (Lapachol). Phytother. Res. 15, 44–48. doi: 10.1002/1099-1573(200102)15:1<44::AID-PTR685>3.0.CO;2-1 11180522

[B75] van de Wall S. Walczak M. van Rooij N. Hoogeboom B. N. Meijerhof T. Nijman H. W.. (2015). Tattoo delivery of a semliki forest virus-based vaccine encoding human papillomavirus E6 and E7. Vaccines (Basel) 3, 221–238. doi: 10.3390/vaccines3020221 26343186 PMC4494346

[B76] Wei X. Li M. Ma M. Jia H. Zhang Y. Kang W.. (2017). Induction of apoptosis by FFJ-5, a novel naphthoquinone compound, occurs *via* downregulation of PKM2 in A549 and HepG2 cells. Oncol. Lett. 13, 791–799. doi: 10.3892/ol.2016.5522 28356960 PMC5351257

[B77] World Health Organization (2022) Leishmaniasis. world health organization. Available at: http://www.who.int/leishmaniasis/en/.

[B78] Yang X. Zhao Y. Chen X. Jiang B. Sun D. (2013). The protective effect of recombinant *Lactococcus lactis oral* vaccine on a *Clostridium difficile*-infectedanimal model. BMC Gastroenterologia 13, 1–13. doi: 10.1186/2046-1682-4-13 PMC3750240 23865596

[B79] Yetisen A. K. Moreddu R. Seifi S. Jiang N. Vega K. Dong X.. (2019). Dermal tattoo biosensors for colorimetric metabolite detection. Angewandte Chemie - Int. Edition 58, 10506–10513. doi: 10.1002/anie.201904416 31157485

[B80] Yigit B. Kabul Gurbulak E. Ton Eryilmaz O. (2022). Usefulness of endoscopic tattooing before neoadjuvant therapy in patients with clinical complete response in locally advanced rectal cancer for providing a safe distal surgical margin. J. Laparoendoscopic Advanced Surg. Techniques 32, 506–514. doi: 10.1089/lap.2021.0382 34232787

[B81] Zulfiqar B. Shelper T. B. Avery V. M. (2017). Leishmaniasis drug discovery: recent progress and challenges in assay development. Drug Discovery Today 22, 1516–1531. doi: 10.1016/j.drudis.2017.06.004 28647378

[B82] Zu X. Xie X. Zhang Y. Liu K. Bode A. M. Dong Z.. (2019). Lapachol is a novel ribosomal protein S6 kinase 2 inhibitor that suppresses growth and induces intrinsic apoptosis in esophageal squamous cell carcinoma cells. Phytother. Res. 33, 2337–2346. doi: 10.1002/ptr.6415 31225674

